# Sedentary behavior in Brazilian children and adolescents: a systematic review

**DOI:** 10.1590/S1518-8787.2016050006307

**Published:** 2016-03-10

**Authors:** Paulo Henrique Guerra, José Cazuza de Farias, Alex Antonio Florindo

**Affiliations:** I Escola de Artes, Ciências e Humanidades. Universidade de São Paulo. São Paulo, SP, Brasil; II Grupo de Estudos e Pesquisas Epidemiológicas em Atividade Física e Saúde. Universidade de São Paulo. São Paulo, SP, Brasil; III Departamento de Educação Física. Centro de Ciências da Saúde. Universidade Federal da Paraíba. João Pessoa, PB, Brasil

**Keywords:** Child, Adolescent, Sedentary Lifestyle, Risk Factors, Review

## Abstract

**OBJECTIVE:**

To describe the methodological characteristics of the studies selected and assess variables associated with sedentary behavior in Brazilian children and adolescents.

**METHODS:**

For this systematic review, we searched four electronic databases: PubMed, Web of Knowledge, LILACS, SciELO. Also, electronic searches were applied in Google Scholar. A supplementary search was conducted in the references lists of the included articles and in non-indexed journals. We included observational studies with children and adolescents aged from three to 19 years developed in Brazil, presenting analyses of associations based on regression methods and published until September 30, 2014.

**RESULTS:**

Of the 255 potential references retrieved by the searches, 49 met the inclusion criteria and composed the descriptive synthesis. In this set, we identified a great number of cross-sectional studies (n = 43; 88.0%) and high methodological variability on the types of sedentary behavior assessed, measurement tools and cut-off points used. The variables most often associated with sedentary behavior were “high levels of body weight” (in 15 out of 27 studies; 55.0%) and “lower level of physical activity” (in eight out of 16 studies; 50.0%).

**CONCLUSIONS:**

The findings of this review raise the following demands to the Brazilian agenda of sedentary behavior research geared to children and adolescents: development of longitudinal studies, validation of measuring tools, establishment of risk cut-offs, measurement of sedentary behavior beyond screen time and use of objective measures in addition to questionnaires. In the articles available, the associations between sedentary behavior with “high levels of body weight” and “low levels of physical activity” were observed in different regions of Brazil.

## INTRODUCTION

Sedentary behavior represents activities of little movement, which occur with the body in sitting or reclining position, and present energy expenditure close to that observed in the resting state (< 1.5MET)^1^ ^,^ ^50^. Nowadays, it comprehends activities present in a big part of everyday life, whether in people’s leisure time (talking to friends, using the phone, watching television, using computers or videogames), in transit (driving, riding public transportation, standing up), or even in environments such as work and school, where people are exposed to longer periods sitting down. A growing body of evidence strengthens the consensus that sedentary behavior is a different domain from physical activity, no longer characterized by its absence, with its own related and determinant factors and implications for health^10^ ^,^ ^32^ ^,^ ^35^.

In children and adolescents, sedentary behavior has usually been represented by exposure to screen-related behaviors, which include measures (separate or unified) of time spent with television, video games, tablets, cell phones and computers^51^ ^,^ ^70^. These, in turn, represent only a fraction of the total time spent by young people with sedentary behaviors, excluding other sedentary activities such as time sitting at school and in transit, for example. However, despite this limitation, the *Pesquisa Nacional de Saúde do Escolar* (PeNSE – Brazilian National School-Based Health Survey) showed that the prevalence of adolescents exposed to at least two hours a day of television is high all over the country (78.0% in total, 79.2% in the female sex, and 76.7% in the male sex)^46^.

A systematic review points out that two or more hours of television a day are associated with various harms to health such as high levels of body weight, decreased physical fitness, low self-esteem scores and worsening of student performance^70^. However, this evidence should be interpreted with caution, since it is heavily based on the results of cross-sectional studies conducted in high-income countries.

Although a recent study has identified a large number of Brazilian publications reporting associated and determinant factors of physical activity and sedentary behaviors in different stages of life^53^, it did not further develop what are the variables frequently associated with these two behaviors in childhood and adolescence. Identifying the factors associated with the adoption of these behaviors is crucial to recognize them and foster the development of preventive measures. It also enables expanding the evidence about the possible implications of long-term exposure to these behaviors for teenager health.

The objectives of this study were: (i) to describe methodological aspects used in Brazilian studies involving the types of sedentary behavior most often assessed, the tools used to assess it and the cut-off points adopted for its classification and (ii) to summarize the variables associated with sedentary behavior in Brazilian children and adolescents.

## METHODS

This study is part of the project “Systematic review of determinants and factors associated with sedentary behavior in children and adolescents”, registered on the International Prospective Register of Systematic Reviews database (PROSPERO-CRD42014014107). Its report is in agreement with the Preferred Reporting Items for Systematic Reviews (PRISMA)^40^.

To compose the synthesis, we searched for scientific articles that adequately met the following criteria: (i) observational studies (cross-sectional, cohort and cases-control); (ii) developed in the Brazilian territory, regardless of their representativeness (local, regional, national); (iii) with results of associations based in regression methods; (iv) reporting measures of sedentary behavior, either by total or type-specific exposure (e.g., screen-related behavior), domain (e.g., leisure, transit, school) or a combination (e.g., time sitting in school and leisure), regardless if evaluated as exposure or outcome variable, and (v) involving child or adolescent samples in the range of three to 19 years of age, or mean age within this range. We have excluded studies that used the term sedentary as a synonym for lack of physical activity (or insufficient physical activity) or those related to special groups (e.g., people with hypertension and diabetes). Since this review was outlined with descriptive purposes and, since its inception, no meta-analysis has been planned, we decided to include separate publications in the same sample, as long as all the inclusion criteria were met.

Regarding the operational process, a researcher conducted the stages of reading and evaluating titles, abstracts and full texts, extracting data and making the synthesis. In view of the descriptive characteristic of this review, the risk of bias of each article was not assessed.

The relevant articles were searched in different ways. Four electronic databases were searched: PubMed, Web of Knowledge, LILACS and SciELO, using the terms and keywords: sedentary behavior OR screen time OR TV time OR sitting time AND factors OR correlates OR determinants AND Brazil OR Brasil AND infant OR child OR adolescent. Also, Google Scholar was searched, with the following search strategy: *comportamento sedentário* (sedentary behavior), *tempo de tela* (screen time) and *Brasil* (Brazil). To prevent the loss of relevant information, we conducted additional searches in the Lattes curriculum (lattes.cnpq.br) of some of the leading researchers on the topic, in the reference lists of the included papers and in non-indexed journals.

Data were extracted in a spreadsheet in which the information had been divided into three domains: (i) descriptive data (location of study, year of collection, sample size, age range, sex); (ii) methods (type of study, selection of participants, sampling unit, exposure, sedentary behavior assessment tool, regression model, adjusted variables and outcome measure) and (iii) results, retrieving the analyses of possible associations between sedentary behavior and variables grouped and organized into five sub-domains: harms to health (e.g., body weight, blood pressure and insulin resistance); environmental (e.g., place of residence); socioeconomic and demographic (e.g., sex, age, skin color and income); behavioral (e.g., sleep time, physical activity, consumption of fruits and vegetables); and occupational (e.g., study hours, work).

The results of the studies were classified into conditions or groups: (i) without statistically significant association (p > 0.05); or with statistically significant association (p < 0.05), either positive (ii) or negative (iii). Positive associations indicate that higher levels or greater amount of time in sedentary behaviors are associated with higher levels of the variable. Negative associations indicate that higher levels or greater amount of time spent in sedentary behaviors are associated with lower levels of the variable.

## RESULTS

After exclusion of the duplicates among the four databases (n = 11), the procedure of systematic searches retrieved 255 references. After assessment of titles and abstracts and considering nine articles retrieved in references lists, 88 references were conduced to full text assessment. With 39 exclusions (no use of regression models: n = 18; adoption of the term sedentary behavior as absence of physical activity: n = 17; no assessment of the sedentary behavior: n = 2; age outside the range of three to 19 years: n = 1; research conducted outside Brazil: n = 1), were considered 49 articles for the descriptive synthesis (Figure).

**Figure:**
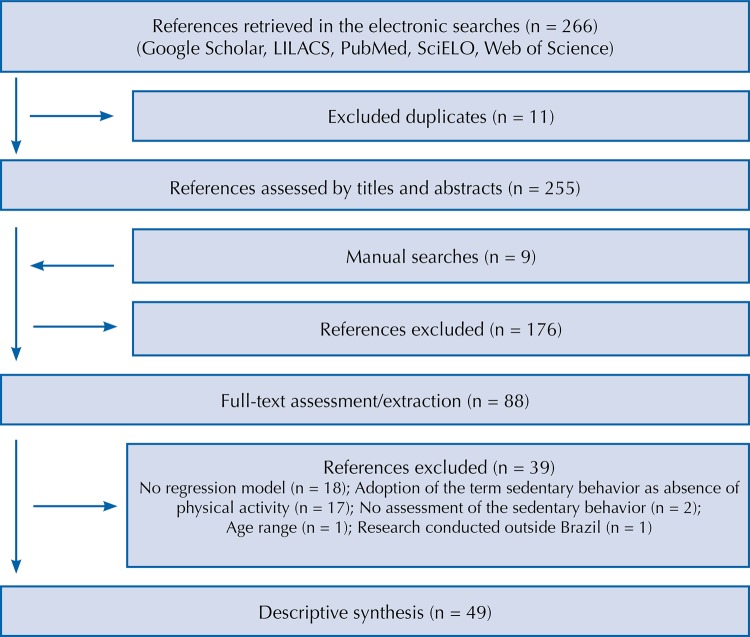
Flowchart of the systematic review.

In total, we observed 38 separate samples in the 49 articles that composed the synthesis, since some of them assessed the same samples (the states of Santa Catarina^63^ ^,^ ^64^ and Pernambuco^68^ ^,^ ^69^ and the cities: Joao Pessoa^61^ ^,^ ^62^, Maringa^13^ ^-^ ^16^, Pelotas^20^ ^-^ ^22^ ^,^ ^24^, and Presidente Prudente^29^ ^-^ ^31^). The samples ranged between 276^12^ and 109,104^56^ participants, with a higher percentage of girls in 23 of them (60.5%). Despite the distinction between the number of articles included in the synthesis and the total number of samples found, we based all analyses of this review on the set of 49 included articles, respecting its descriptive character.

Also in Table 1, by geographic location, we observed a predominance of articles produced in the Southern region of the Country (n = 20; 40.8%), followed by the ones developed in the Southeast and Northeast, with 11 each (22.4% for each region). In this set, we point out two publications of national representativity, based on data from PeNSE, involving adolescents of all state capitals and the Federal District^7^ ^,^ ^56^. Only eight articles had data collection deadlines prior to the year 2005 (16.3%), and sedentary behavior was the outcome variable in 13 articles (26.5%).

**Table 1:** Descriptive characteristics Var-Of studies included.

Reference	Location (year of collection)	Sample	Age	%F	Var
Alves et al.^2^ (2012)	Salvador, BA (2006)	803	10-14	50.6	E
Balaban et al.^4^ (2010)	Juazeiro do Norte, Recife, PE and Sao Paulo, SP (2003-2004)	366	2-6	51.9	E
Beck et al.^5^ (2014)	Tres de Maio, RS (2006)	660	14-19	52.0	E
Camelo et al.^7^ (2012)	All state capitals of the Country (2009)	59,809	Ninth grade SS	52.7	O
Campagnolo et al.^8^ (2008)	Sao Leopoldo, RS (2002, 2003)	810	10-19	59.4	E
Costa et al.^11^ (2011)	Florianopolis, SC (2002)	2,195	7-10	48.8	E
Crispim et al.^12^ (2013)	Goiania, GO (2011-2012)	276	2-4	47.5	E
de Moraes et al.^13^ (2009)	Maringa, PR (2007)^a^	991	14-18	54.5	E
de Moraes et al.^14^ (2012)	Maringa, PR (2007)^a^	991	14-18	54.5	E
de Moraes; Falcão^15^ (2013)	Maringa, PR (2007)^a^	991	14-18	54.5	E
de Moraes et al.^16^ (2013)	Maringa, PR (2007)^a^	991	14-18	54.5	E
de Vitta et al.^17^ (2011)	Bauru, SP (2007)	1,236	11-14	51.8	E
de Vitta et al.^18^ (2014)	Bauru, SP (2009)	524	Fifth to eight grade SS	46.9	E
Dias et al.^19^ (2014)	Cuiaba, MT (2009-2011)	1,716	10-17	49.3	O
Dumith et al.^20^ (2010)	Pelotas, RS (2004-2005)^b^	4,431	11	ND	O
Dumith et al.^21^ (2012)	Pelotas, RS (2008)^b^	4,118	15	49.9	O
Dumith et al.^22^ (2012)	Pelotas, RS (2008)^b^	4,120	15	49.9	E
Duncan et al.^23^ (2011)	Western region of the state of Sao Paulo (nd)	3,397	7-18	53.0	E
Duquia et al.^24^ (2008)	Pelotas, RS (2004, 2005)^b^	4,452	11	50.8	E
Dutra et al.^25^ (2006)	Pelotas, RS (2003)	810	10-19	49.7	E
Enes; Slater^26^ (2013)	Piracicaba, SP (2004-2005)	431	10-13	56.0	E
Faria et al.^27^ (2014)	Vicosa, MG (nd)	800	10-19	51.2	E
Farias Jr. et al.^28^ (2012)	Joao Pessoa, PB (2009)	2,859	14-19	57.8	E
Fernandes et al.^29^ (2008)	Presidente Prudente, SP (2007)^c^	1,752	11-17	53.7	E
Fernandes et al.^30^ (2011)	Presidente Prudente, SP (2007)^c^	1,630	11-17	54.0	O
Fernandes et al.^31^ (2011)	Presidente Prudente, SP (2007)^c^	1,779	11-17	ND	E
Guimarães et al.^36^ (2013)	Curitiba, PR (2012)	572	12-17	57.0	E
Hackenhaar et al.^37^ (2013)	Cuiaba, MT (2009-2011)	1,716	10-17	50.7	E
Lippo et al.^41^ (2010)	Recife, PE (nd)	597	15-19	49.4	E
Melo et al.^45^ (2011)	State of Pernambuco (2010)	4,207	14-19	59.8	O
Oliveira et al.^49^ (2010)	Sao Luis, MA (2005)	592	9-16	50.5	O
Petribú et al.^52^ (2011)	Caruaru, PE (2007)	600	15-20	62.5	E
Rech et al.^54^ (2010)	Morro Reuter and Dois Irmaos, RS (2005)	1,442	7-12	50.0	E
Rech et al.^55^ (2013)	Caxias do Sul, RS (2011)	1,230	11-14	49.3	E
Rezende et al.^56^ (2014)	All state capitals of the Country (2012)	109,104	Ninth grade SS	ND	E
Ribeiro et al.^57^ (2003)	Sao Paulo, SP (2000)	446	7-10	52.0	E
Santos et al.^58^ (2013)	Uberaba, MG (2012)	649	9-12	52.1	O
Silva et al.^59^ (2011)	Florianopolis, SC (2007)	818	14-18	61.8	E
Silva Jr. et al.^60^ (2012)	Rio Branco, AC (2009)	741	14-18	54.1	E
Silva et al.^61^ (2007)	Joao Pessoa, PB (2005)d	1,570	7-12	48.5	O
Silva et al.^62^ (2007)	Joao Pessoa, PB (2005)d	1,570	7-12	48.5	E
Silva et al.^63^ (2008)	State of Santa Catarina (2001)^e^	5,028	15-19	59.4	E
Silva et al.^64^ (2009)	State of Santa Catarina (2001-2002)^e^	5,028	15-19	59.4	O
Silva et al.^65^ (2014)	State of Santa Catarina (2011)	6,529	15-19	57.8	O
Smith-Menezes et al.^66^ (2012)	Aracaju, SE (2007)	758	18	0	E
Suñé et al.^67^ (2006)	Capao da Canoa, RS (2004)	719	11-13	ND	E
Tassitano et al.^68^ (2009)	State of Pernambuco (2006)^f^	4,210	14-19	59.8	E
Tenório et al.^69^ (2010)	State of Pernambuco (2006)^f^	4,210	15-19	59.8	O
Vasconcellos et al.^71^ (2013)	Niteroi, RJ (2010)	328	10-18	67.1	E

%F: percentage of girls in the sample; O: outcome variable; E: exposure variable; SS: secondary school; ND: not described; Var: variable (how the sedentary behavior was analyzed)

^a-f^ Publications that used similar samples.

The synthesis of this review was based on 43 cross-sectional studies, three case-control studies^4^ ^,^ ^41^ ^,^ ^57^, and three studies from the Pelotas cohort^21^ ^,^ ^22^ ^,^ ^26^ (Table 2). About the measurement of sedentary behavior, observations of screen time (n = 27; 55.1%) and television time (n = 16; 32.6%) exposures prevailed. The cut-off point most often used in the articles to characterize excess of time in sedentary behaviors was at least two hours a day, adopted in 16 publications (30.6%). It comprehends: screen time (n = 10); time watching television (n = 4); time using the computer (n = 1); or screen time (n = 1). Even if all the articles included have used questionnaires to assess sedentary behavior, there was great variation in the tools used. In 17 articles (34.7%), the authors did not report if the tool had been validated or, in the case of foreign tools, if it had been validated for use in Brazilian populations.

**Table 2:** Methodological characteristics of studies included.

Reference	Type	Selection	Cut-off points	Assessment tool	Regression model/ Effect measure
Alves et al.^2^ (2012)	CS	ran	Screen > 3.3h/week	QDS^a^ ^,b^	Poisson/PR
Balaban et al.^4^ (2010)	CC	conv	TV > 5h/d	QDS^c,d^	Logistic/OR
Beck et al.^5^ (2014)	CS	ran	Screen h/week	QDS^d^	Linear/Coef.
Camelo et al.^7^ (2012)	CS	ran	TV > 2h/d	2008 PeNSE	Logistic/OR
Campagnolo et al.^8^ (2008)	CS	ran	TV > 2h/d	7-Day Recall^d^	Poisson/PR
Costa et al.^11^ (2011)	CS	ran	Screen > 2h/d	QDS	Poisson/PR
Crispim et al.^12^ (2013)	CS	ran	TV > 2h/d	QDS^c,d^	Poisson/PR
de Moraes et al.^13^ (2009)	CS	ran	Screen > 4h/d	IPAQ-short	Poisson/PR
de Moraes et al.^14^ (2012)	CS	ran	Screen h/d	QDS	Linear/Coef.
de Moraes; Falcão.^15^ (2013)	CS	ran	Screen > 4h/d	QDS	Poisson/PR
de Moraes et al.^16^ (2013)	CS	ran	Screen > 2h/d	QDS	Linear/Coef.
de Vitta et al.^17^ (2011)	CS	ran	Screen > 2h/d	QDS^d,e^	Logistic/OR
de Vitta et al.^18^ (2014)	CS	ran	Screen h/d	QDS^d,e^	Logistic/OR
Dias et al.^19^ (2014)	CS	ran	Screen > 4h/d	QDS^d^	Logistic/OR
Dumith et al.^20^ (2010)	CS-CO	1993 birth	Screen > 2h/d	QDS	Poisson/PR
Dumith et al.^21^ (2012)	CO	1993 birth	Screen Frequency	QDS	Linear/Coef.
Dumith et al.^22^ (2012)	CO	1993 birth	Screen > 2h/d	QDS	Poisson/RR
Duncan et al.^23^ (2011)	CS	ran	PC h/d	QDS	Logistic/OR
Duquia et al.^24^ (2008)	CS-CO	1993 birth	Screen > 4h/d	QDS	Poisson/PR
Dutra et al.^25^ (2006)	CS	ran	TV > 4h/d	QDS	Poisson/PR
Enes; Slater^26^ (2013)	CO	ran	Screen h/d	Berkey questionnaire^f^	Linear/Coef.
Faria et al.^27^ (2014)	CS	conv	Sitting min/d	IPAQ-short	Logistic/OR
Farias Jr. et al.^28^ (2012)	CS	ran	Screen > 2h/d	QDS^d^	Poisson/PR
Fernandes et al.^29^ (2008)	CS	ran	TV Freq	Baecke questionnaire^f^	Poisson/PR
Fernandes et al.^30^ (2011)	CS	ran	TV Freq	Baecke questionnaire^f^	Logistic/OR
Fernandes et al.^31^ (2011)	CS	ran	TV Freq	Baecke questionnaire^f^	Poisson/PR
Guimarães et al.^36^ (2013)	CS	conv	Total	ASAQ	Logistic/OR
Hackenhaar et al.^37^ (2013)	CS	conv	Screen > 4h/d	COMPAC questionnaire	Poisson/PR
Lippo et al.^41^ (2010)	CC	conv	Screen > 1h/d	QDS^d^	Logistic/OR
Melo et al.^45^ (2011)	CS	ran	TV > 3h/d	GSHS-WHO	Logistic/OR
Oliveira et al.^49^ (2010)	CS	ran	Screen > 2h/d	24h PA recall^g^	Linear/Coef.
Petribú et al.^52^ (2011)	CS	ran	TV > 3h/d	COMPAC questionnaire	Logistic/PR
Rech et al.^54^ (2010)	CS	conv	TV > 3h/d	QDS	Logistic/PR
Rech et al.^55^ (2013)	CS	ran	Screen > 3h/d	QDS^d^	Logistic/PR
Rezende et al.^56^ (2014)	CS	ran	Screen + Sitting 2h/d	2012 PeNSE	Poisson/PR
Ribeiro et al.^57^ (2003)	CC	ran	TV > 4h/d	QDS^c^	Logistic/OR
Santos et al.^58^ (2013)	CS	ran	Screen frequency	Lifestyle questionnaire	Poisson/PR
Silva et al.^59^ (2011)	CS	ran	TV > 2h/d	QDS^d^	Logistic/OR
Silva Jr. et al.^60^ (2012)	CS	ran	PC > 2h/d	IPAQ-short	Poisson/PR
Silva et al.^61^ (2007)	CS	ran	TV Freq	QDS	Logistic/OR
Silva et al.^62^ (2007)	CS	ran	Screen h/d	QDS	Poisson/PR
Silva et al.^63^ (2008)	CS	ran	Screen > 2h/d	COMPAC questionnaire	Logistic/OR
Silva et al.^64^ (2009)	CS	ran	Screen > 4h/d	COMPAC questionnaire	Poisson/PR
Silva et al.^65^ (2014)	CS	ran	Screen > 2h/d	COMPAC questionnaire	Poisson/PR
Smith-Menezes et al.^66^ (2012)	CS	ran	Screen > 2h/d	IPAQ-short	Poisson/PR
Suñé et al.^67^ (2006)	CS	ran	Total > 4h 30min	QDS^d^	Poisson/PR
Tassitano et al.^68^ (2009)	CS	ran	TV > 3h/d	GSHS-WHO	Logistic/OR
Tenório et al.^69^ (2010)	CS	ran	TV > 3h/d	GSHS-WHO	Logistic/OR
Vasconcellos et al.^71^ (2013)	CS	ran	Screen h/week	Pate et al. questionnaire^h^	Logistic/OR

CS: cross-sectional; ran: random selection; Screen: screen activities such as those with television, computer and video games; h/week: hours per week; QDS: questionnaire developed for the study; PR: prevalence ratio; CC: case-control; conv: convenience sampling; TV: television; h/d: hours per day; OR: odds ratio; Coef.: coefficient; PeNSE: Brazilian National School-Based Health Survey; PC: computer; RR: relative risk; IPAQ: International Physical Activity Questionnaire; CS-CO: cross-sectional analysis in a cohort study; CO: cohort; Freq: frequency; min/d: minutes per day; QASA: Adolescent Sedentary Activity Questionnaire; COMPAC: Health risk behaviors in youths of the Santa Catarina state project (*Comportamento do Adolescente Catarinense*)

^a^ Adapted from the Global School-Based Student Health Survey – World Health Organization (GSHS-WHO).

^b^ Adapted from the Pro Children Cross-sectional Survey (CSS).

^c^ Proxy approach.

^d^ The article does not report prior validation of the tool (or does not report international tool validation in Brazilian populations).

^e^ Based on the study by Harreby et al., “Risk factors for low back pain in a cohort of 1389 Danish school children: an epidemiologic study” (*Eur Spine J* 1999;8:444).

^f^ Based on the study of Berkey et al., “Activity, Dietary Intake, and Weight Changes in a Longitudinal Study of Preadolescent and Adolescent Boys and Girls” (*Pediatrics* 2000,105:e56).

^g^ Based on the Self-Administred Physical Activity Checklist.

^h^ Translated and validated by Barros; Nahas, 2003 in *“Medidas da atividade física: teoria e aplicação em diversos grupos populacionais”*.

The articles presented analyses between sedentary behavior and 31 different variables (Table 3). Most of these have been classified as behavioral variables (n = 12). The large number of variables belonging to demographic or socioeconomic domains (n = 7) and harms to health (n = 6) is also noteworthy.

**Table 3:** Synthesis of the relationships between the variables and high volumes of sedentary behavior in Brazilian children and adolescents.

Domains and variables	Association as a risk factor	Association as a protective factor	No association
Socioeconomic			
High socioeconomic status	Screen time:^19^ ^,^ ^20^; PC+VG:^65^	TV time:^65 (F)^	Screen time:^49,58,64,66^; TV:^31^
Skin color	–	–	Screen time:^20^; TV:^69^
Age	Screen time:^19 (association with increasing age)^	TV time:^65,69 (association with age range from 17 to 19 years)^ PC+VG time:^65 (association with age range from 17 to 19 years)^	Screen time:^49,58,64^
Living with parents	–	–	TV time:^69^
More educated mother	–	–	TV time:^69^
Education	–	–	Screen time:^66^
Sex	–	TV time:^69 (F)^	Screen time:^20,58^; TV time:^69 (SEM)^
Environmental			
Liking the neighborhood in which they live	–	–	Screen time:^20^
Activity place^a^	–	–	Screen time:^20^
Place of residence	PC+VG time:^65 (association with living in urban areas)^	Screen time:^19 (association with living in inland towns), 65 (association with living in rural areas)^; TV time:^65 (F; association with living in urban areas), 69 (association with living in rural areas)^	Screen time:^64^; TV time:^69^
Behavioral			
Perception of well-being	Screen time:^20 (adolescents who reported higher levels of screen time were not classified at the highest level of happiness)^	–	–
Sleep time	–	–	Screen time:^58^
Religious affiliation	TV time:^45 (high levels of TV time were observed in people of Catholic religion, WE)^	–	TV time: ^45 (no association found between type of religion and high levels of TV, WEEK)^
Religious practice	–	TV time:^45 (religious practice showed to be a protective factor against high levels of TV time)^	–
Sports^b^	–	–	TV time:^29 (engaging in sports)^; Screen time:^58 (attending a sports school)^
Lower level of physical activity	Screen time + Total time sitting down:^56 (LPA)^; Screen time:^19 (300 minutes per week), 21 (LPA), 22 (LPA, F), 63 (300 minutes per week, M)^; TV time:^41 (IPAQ)^	TV time:^11 (protection for children with levels of less than 2h/day of TV by DAFA), 30 (protection for girls with levels of less than 2h/day of TV on WE)^	Screen time:^13,20,22,28,58,63 (F)^; TV time:^2^; PC time:^41^
Experimentation with alcohol	Screen time:^19^	–	–
Marital status	–	–	Screen time:^66^
Transit to school	–	–	Screen time:^61^
Fruit and vegetable consumption	–	–	Screen time:^64^; TV time:^30^
Bullying (victim and aggressor)	Screen time:^55^	–	–
Foods and beverages of high energy content	Screen time:^14 (F protein consumption)^; TV time:^7,30^	–	Screen time:^14(F consumption of foods of high energy content)^
Occupational			
Total dismissal from or little participation in PE classes	–	–	Screen time + Total time sitting down:^56^; TV time:^69^
Work	–	–	Screen time:^64,66^
School session	–	–	Screen time:^64^
Harms to health			
Blood pressure	–	–	Screen time:^16,21,58^; TV time:^12^
Total cholesterol	Screen time:^36^	–	Total time:^36^; Screen time:^5^
Musculoskeletal pain (neck or shoulders)	TV time:^18^; PC time:^18 (M)^	–	PC time:^18 (F)^
Lower back pain	Screen time:^17^	–	–
High levels of body weight	Total time:^36,67,71^; Screen time:^19 (adolescence),20,21,37^; TV time:^8,25,52,54,57,62 (M)^; PC time:^23,60^	–	Screen time:^15,19 (childhood),24,26,36,58,63^; TV time:^4,31,59,61 (F),68^
Insulin resistance	Total time sitting down:^27^	–	–

PC: computer; VG: videogames; TV: television; F: female; WEEK: days of the week; LPA: leisure time physical activity; M: male; IPAQ: classification of moderate and vigorous physical activity according to the International Physical Activity Questionnaire (IPAQ); DAFA: physical activity level classification (light, moderate and vigorous) according to the Physical Activity and Nutrition Typical Day (*Dia Típico de Atividade Física e Alimentação*) questionnaire; WE: days of the weekend; Total time: total time in sedentary behaviors; PE: Physical education

^a^ Relationships between sedentary behavior and daily activities in enclosed (e.g., home) or open (e.g., parks) spaces.

^b^ Relationships between levels of sedentary behavior and sports, as well as enrollment in sports schools.

As for the number of articles and the frequency of associations between the measurement of sedentary behavior and the variables of one or more domains, the main result found was the positive association between high volumes of sedentary behavior and “high levels of body weight”, observed in 15 of the 27 articles that evaluated this relationship (55.5%). Most of the results that identified statistically positive associations between sedentary behavior and that variable were from studies that assessed screen behaviors, whether specific (such as television and computer) or total screen time. On the measurement effect of these studies, eight statistically positive associations were expressed by prevalence ratios, six by odds ratios and one by coefficient (β). We point out that two of those positive associations between higher volumes of sedentary behavior and “high levels of body weight” derive from longitudinal studies (one cohort and one case-control) (Table 3).

As a secondary result, 50.0% (eight of 16) of articles have shown statistically positive associations between high volumes of sedentary behavior and “low levels of physical activity”. As with the previous result, most of those studies evaluated sedentary behavior by screen behaviors (total, n = 4, and television, n = 3). By type of study, we observed two of those positive associations in the studies based on the 1993 Pelotas birth cohort^,^ ^21^ ^,^ ^22^ (Table 3).

Even if present in only three articles each, two other observations should be considered. The first is the positive associations between longer screen time and the consumption of foods rich in energy and fats (such as snacks, sweet biscuits, sausages and cold meats, and drinks of high energy density)^7^ ^,^ ^14^ ^,^ ^30^. The other is the protective associations between living in inland towns or rural areas and sedentary behavior^19^ ^,^ ^64^ ^,^ ^69^. On the other hand, due to the limited number of articles and similar distribution between the presence or absence of statistically positive associations, there still is uncertainty as to the possible associations between high levels of sedentary behavior and the variables “high socioeconomic status” (n = 9 articles), “age” (n = 7 articles) and “sex” (n = 3 articles) (Table 3).

## DISCUSSION

The main results of this systematic review come from the data of 49 articles involving samples of Brazilian child and adolescent populations. In this set, we highlight the large number of cross-sectional studies, as well as the great methodological discrepancy among the articles as to the types of sedentary behaviors evaluated and the cut-off points used to characterize excess of time spent in sedentary behaviors. The analyzed articles showed greater consistency in the statistically positive associations between long exposure time to sedentary behavior and two variables: (i) high levels of body weight and (ii) low levels of physical activity.

On the methodological aspects, the present study identified the lack of standards in the assessment of sedentary behavior in children and adolescents, either for different ways to characterize these behaviors (e.g., screen time, time sitting down, television time) or for the methods and tools used in its assessment (international questionnaires, national questionnaires, or even questionnaires developed specially for those studies)^9^ ^,^ ^51^. Since all the studies used questionnaires to measure sedentary behavior, one of the main challenges for future research is adding objective measurements in its methods (e.g., by motion sensors, such as ActiPal and accelerometers) to complement the self-reported data, controlling the memory bias, as observed in a study with adults^39^.

Twelve publications (24.5%) did not report any evidence of validation of the tool used. These findings reinforce the observation of Atkin et al.^3^, who highlight the lack of good tools to assess sedentary behavior in epidemiological research. It is important that future studies, in addition to reporting the validation process of their questionnaires or presenting references that support such procedure, use validated questionnaires.

Corroborating the findings of a prior review^70^, most studies found characterize sedentary behavior as a measurement of television time or screen time, which combines the indicators of time spent watching television, using the computer and playing video games. However, these behaviors represent only part of the total time that children and adolescents spend in sedentary behavior throughout the day while they are awake. In addition, the studies included in this review showed high heterogeneity among the cut-off points adopted to define the excessive time of exposure to sedentary behavior. The cut-off point of two hours a day was the most frequent between the included studies, used in 16 articles. Even though Brazilian studies have not clarified the health risks of the amount of time spent in sedentary behaviors, a systematic review of international studies identified statistically significant associations between exposures of at least two hours and overweight, low physical fitness, low self-esteem and socialization, and low academic performance^70^.

About the regression models adopted, 22 articles (21 cross-sectional and one cohort study) used Poisson regression and 21 articles (19 cross-sectional and two case-control studies) used logistic regression. In six articles (four cross-sectional and two cohorts), analyses were performed using the linear regression model.

The positive associations observed between increased sedentary behavior time and increased levels of body weight corroborate the syntheses of reviews involving children and adolescents from different countries, regardless of their socioeconomic status^43^ ^,^ ^47^, and a synthesis of prospective studies conducted in high income countries such as Australia, Canada and the United States of America^48^. As seen in this article, it is important to point out that this evidence is largely based on the results of articles assessing screen behaviors, with a particular focus on television time.

The positive associations observed between longer time in sedentary behavior and low levels of physical activity agree with a study conducted in high-income countries that showed negative relations between high screen time and moderate and vigorous physical activities^44^. The main recommendation of physical activity for health gains advocates moderate and vigorous intensity, disregarding light physical activities^a^. Stronger inverse correlations have been found between sedentary behavior and light physical activities^39^. To avoid long uninterrupted periods of sedentary behavior, constant breaks are proposed during activities characterized as sedentary (< 1.5 MET), by inserting activities of higher energy expenditure^38^. Even though a recent meta-analysis pointed out the potential for this strategy with children and adolescents in the school environment^33^, none of the interventions included were conducted in Brazil, which shows the need for these studies in the national research agenda.

Although observed in fewer publications, we found positive associations between more time spent in sedentary behavior and consumption of food and beverages of high energy content as well as positive associations between less time spent in sedentary behavior and living in inland towns or rural areas. In relation to the first result, this one’s data corroborate the findings of recent international studies that have shown associations between long screen time (≥ 20 hours per week) and high consumption of foods of high energy content (snacks, sweets and biscuits) and soft drinks^6^ ^,^ ^34^ ^,^ ^42^. Regarding the protective effect of living in inland towns or in rural areas, it is believed that children and adolescents who live in these places can have better opportunities for physical activity. The more favorable safety and traffic conditions would enable playing more freely in the communities and also using more bicycles^19^ ^,^ ^64^. This would also reduce sedentary behaviors as screen activities in the leisure time and television watching for long periods. However, as the number of available national studies that assessed the occurrence of these associations is still small, more research is needed.

This systematic review showed some gaps among Brazilian studies about sedentary behavior such as the high number of cross-sectional studies included compared with the limited number of longitudinal studies^53^. As the cross-sectional study design does not establish antecedence between exposure and outcome variables, reverse causation can occur. This is the case of the main study finding: it is impossible to know the chronological sequence between sedentary behaviors and body weight and physical activity bevels in children and adolescents. In addition, the lack of longitudinal studies limits inferences about the dose-response relationships between sedentary behavior and the variables found. Another limitation was the high heterogeneity of the included articles in the forms of assessing sedentary behavior (screen, television, computer, seated, and total time), as the cut-off points adopted and the tools used for its evaluation.

The inclusion of all studies with repeated samples can be considered a potential limitation of this systematic review. This decision may have overestimated the results of comparison of the relationships between sedentary behavior and other outcomes or exposures, as high levels of body weight or low levels of physical activity. However, two methodological decisions led to the inclusion of these articles: the descriptive aim of the present review, which sought to provide an overview of the variables associated with sedentary behavior in children and adolescents, and the planning of not conducting a meta-analysis.

Based on the data identified in this review, we can make some conclusions. There are still few studies that measure sedentary behavior beyond screen time (television, computer, video games). New studies may involve measurement of time spent on cell phones and tablets, as well as in other domains, such as sitting down in transit or in the school environment. Another point is the need to use validated questionnaires in conjunction with objective tools to strengthen the information about the association between time spent in sedentary behavior and different outcomes. In view of the predominance of cross-sectional studies, longitudinal studies are needed, especially to establish the chronological sequence of events and the dose-response relationship. On the results available, the studies conducted with samples of Brazilian children and adolescents showed mainly associations between increased sedentary behavior and “high levels of body weight” and “low levels of physical activity” in different regions of Brazil.

## Footnotes

a World Health Organization. *Global recommendations on physical activity for health*. Geneva: World Health Organization; 2010.
