# Supplementary material for: Sedentary behavior in Brazilian children and adolescents: a systematic review
Source: Rev Saude Publica. 2016 Mar 10;50:9. doi: 10.1590/S1518-8787.2016050006307 (PMC4794779; doi:10.1590/S1518-8787.2016050006307)
Supplement: Translation

# Translation: Comportamento sedentário em crianças e adolescentes brasileiros: revisão sistemática

**Authors:** Paulo Henrique Guerra, José Cazuza de Farias, Alex Antonio Florindo

**Affiliations:** I Escola de Artes, Ciências e Humanidades. Universidade de São Paulo. São Paulo, SP, Brasil; II Grupo de Estudos e Pesquisas Epidemiológicas em Atividade Física e Saúde. Universidade de São Paulo. São Paulo, SP, Brasil; III Departamento de Educação Física. Centro de Ciências da Saúde. Universidade Federal da Paraíba. João Pessoa, PB, Brasil

**Keywords:** Criança, Adolescente, Estilo de Vida Sedentário, Fatores de Risco, Revisão

## Abstract

**OBJETIVO:**

Descrever as características metodológicas dos estudos selecionados e verificar variáveis associadas ao comportamento sedentário em crianças e adolescentes brasileiros.

**MÉTODOS:**

Para esta revisão sistemática, foram realizadas buscas nas bases de dados eletrônicas PubMed, *Web of Knowledge*, Lilacs, SciELO e Google Acadêmico. Foi feita busca complementar nas listas de referências dos artigos analisados e em periódicos não indexados. Foram incluídos estudos observacionais com crianças e adolescentes de três a 19 anos de idade desenvolvidos no Brasil, que apresentaram análises de associações por meio de métodos de regressão, publicados até 30 de setembro de 2014.

**RESULTADOS:**

Das 264 referências recuperadas nas buscas, 49 atenderam aos critérios de inclusão e compuseram a síntese descritiva. Neste conjunto, identificou-se grande número de pesquisas transversais (n = 43; 88,0%) e alta variabilidade metodológica sobre os tipos de comportamento sedentário avaliados, instrumentos de avaliação utilizados e pontos de corte adotados. As variáveis mais frequentemente associadas ao comportamento sedentário foram “níveis elevados de peso corporal” (em 15 de 27 estudos; 55,0%) e “menor nível de atividade física” (em oito de 16 estudos; 50,0%).

**CONCLUSÕES:**

Os achados da presente revisão levantam as seguintes demandas à agenda brasileira de pesquisa em comportamento sedentário voltada às crianças e adolescentes: realização de estudos longitudinais, validação de instrumentos de medida, estabelecimento de pontos de corte, mensuração do comportamento sedentário para além do tempo de tela e utilização de medidas objetivas como recurso paralelo ao questionário. Nos artigos disponíveis, as associações entre comportamento sedentário com “níveis elevados de peso corporal” e “baixos níveis de atividade física” foram observadas em distintas regiões do território brasileiro.

## INTRODUÇÃO

Comportamento sedentário representa atividades de pequena movimentação, que ocorrem com o corpo na posição sentada ou reclinada, e têm gasto energético próximo ao observado no estado de repouso (< 1,5MET)^1^ ^,^ ^50^. Na atual caracterização, são englobadas atividades que estão presentes em parte expressiva da vida cotidiana, seja no tempo de lazer, ao conversar com amigos, fazer uso do telefone, assistir à televisão, usar computador ou videogame, seja no deslocamento, ao dirigir, trafegar sentado nos transportes coletivos, ou mesmo em ambientes como o do trabalho e da escola, onde as pessoas estão expostas a jornadas mais ampliadas de tempo sentado. Crescente corpo de evidências fortalece o consenso de que se trata de um comportamento distinto da atividade física, não mais caracterizando-se pela ausência desta, com fatores correlatos e determinantes e implicações para saúde próprios^10^ ^,^ ^32^ ^,^ ^35^.

Em estudos direcionados a crianças e adolescentes, o comportamento sedentário tem sido usualmente representado pela exposição aos comportamentos de tela, que compreendem as medidas (unificadas ou distintas) do tempo de televisão, videogame, *tablets*, aparelhos celulares e computador^51^ ^,^ ^70^. Estes, por sua vez, representam apenas uma parte do tempo total despendido pelos jovens em comportamentos sedentários, excluindo outras atividades sedentárias como o tempo sentado na escola e no deslocamento, por exemplo. Entretanto, apesar dessa delimitação, a Pesquisa Nacional de Saúde do Escolar mostrou que a prevalência de adolescentes expostos a pelo menos duas horas diárias de televisão é alta no Brasil (78,0% no total, sendo 79,2% para o sexo feminino e 76,7% para o sexo masculino)^46^.

Revisão sistemática aponta que o volume diário igual ou superior a duas horas de televisão está associado a diversos malefícios à saúde, como níveis elevados de peso corporal, diminuição da aptidão física, baixos escores de autoestima e piora no desempenho estudantil^70^. No entanto, essa evidência deve ser interpretada com muita cautela, uma vez que está fortemente alicerçada nos resultados de estudos transversais conduzidos em países de renda alta.

Embora pesquisa recente tenha identificado grande número de publicações brasileiras reportando fatores associados e determinantes da atividade física e comportamentos sedentários nas distintas fases da vida^53^, ela não aprofundou quais as variáveis que estão frequentemente associadas a estes dois comportamentos nas fases da infância e adolescência. Identificar os fatores associados à adoção desses comportamentos é essencial para reconhecê-los e, a partir disso, fomentar a elaboração de intervenções preventivas. Ainda, possibilita ampliar as evidências sobre as possíveis implicações da exposição prolongada a esses comportamentos à saúde dos adolescentes.

Os objetivos do presente estudo foram: (i) descrever aspectos metodológicos usados nos estudos nacionais, envolvendo os tipos de comportamento sedentário mais frequentemente avaliados, os instrumentos utilizados para sua medida e os pontos de corte adotados para a sua classificação e (ii) sumarizar as variáveis associadas ao comportamento sedentário em crianças e adolescentes brasileiros.

## MÉTODOS

Este trabalho pertence ao projeto “*Systematic review of determinants and factors associated with sedentary behavior in children and adolescents*”, registrado na base *International Prospective Register of Systematic Reviews* (PROSPERO –CRD42014014107). Seu relato está em concordância com o *Preferred Reporting Items for Systematic Reviews* (PRISMA)^40^.

Para formulação da síntese, procurou-se por artigos científicos que atendessem adequadamente aos seguintes critérios: (i) estudos observacionais (transversais, coortes e casos-controles); (ii) desenvolvidos no território brasileiro, independentemente de sua representatividade (local, regional, nacional); (iii) apresentar resultados de associações baseados em métodos de regressão; (iv) relatar medidas do comportamento sedentário, seja por sua exposição total ou por algum tipo específico (e.g., comportamentos de tela), domínio (e.g., lazer, deslocamento, escola) ou combinação (e.g., tempo sentado na escola e no lazer), independente se avaliado como variável de exposição ou de desfecho e (v) envolver amostras de crianças ou adolescentes na faixa dos três aos 19 anos de idade, ou com média de idade nesse intervalo. Não foram incluídos trabalhos que utilizaram o termo sedentário como sinônimo de ausência de prática de atividade física (ou prática de atividade física insuficiente) ou aqueles relacionados a grupos especiais (e.g., hipertensos e diabéticos). Visto que esta revisão foi delineada com fins exploratórios e não foi, desde seu princípio, planejada a realização de meta-análise, optou-se pela inclusão de publicações distintas com uma mesma amostra, desde que atendidos todos os critérios de inclusão supracitados.

Quanto ao processo operacional da pesquisa, as fases de leitura e avaliação dos títulos, resumos e textos integrais, de extração de dados e de elaboração da síntese foram conduzidas por um pesquisador. Em vista do caráter descritivo desta revisão, não se avaliou o risco de viés de cada artigo.

Os artigos relevantes foram procurados em diferentes meios. Nos meios eletrônicos, foram feitas buscas em quatro bases de dados: PubMed, *Web of Knowledge*, Lilacs e SciELO, usando-se os termos e descritores: *sedentary behavior OR screen time OR TV time OR sitting time AND factors OR correlates OR determinants AND Brazil OR Brasil AND infant OR child OR adolescent.* Também realizou-se buscas no Google Acadêmico, aplicando-se a seguinte estratégia de busca: comportamento sedentário, tempo de tela e Brasil. Para evitar a perda de informação relevante, foram conduzidas buscas complementares nos currículos *Lattes* (lattes.cnpq.br) de alguns dos principais pesquisadores sobre o tema, nas listas de referências dos trabalhos incluídos e em periódicos não indexados.

A extração dos dados foi realizada em planilha eletrônica, na qual as informações foram agrupadas em três domínios: (i) dados descritivos (local do estudo, ano da coleta, tamanho da amostra, faixa etária, sexo); (ii) metodológicos (tipo de estudo, seleção dos participantes, unidade amostral, exposição, instrumento de avaliação do comportamento sedentário, modelo de regressão, variáveis ajustadas e medida de desfecho) e (iii) resultados, recuperando as análises que verificaram possíveis associações entre o comportamento sedentário e variáveis agrupadas e organizadas em cinco subdomínios: agravos à saúde (e.g., peso corporal, pressão arterial sistêmica e resistência à insulina); ambientais (e.g., local de residência); demográficos e socioeconômicos (e.g., sexo, idade, cor da pele e renda); comportamentais (e.g., tempo de sono, prática de atividade física, consumo de frutas e verduras); e ocupacionais (e.g., turno de estudo, trabalho).

Os resultados dos estudos foram classificados em condições ou grupos: (i) sem associação estatisticamente significativa (p > 0,05); ou com associação estatisticamente significativa (p < 0,05), podendo ser positiva (ii) ou negativa (iii). As associações positivas indicam que maiores níveis ou maior quantidade de tempo em comportamentos sedentários estão associados a maiores níveis da variável em análise. As associações negativas indicam que maior quantidade de tempo ou maiores níveis em comportamentos sedentários estão associados a menores níveis da variável em análise.

## RESULTADOS

Excluídas as duplicatas entre as bases de dados (n = 11), o procedimento de buscas eletrônicas recuperou 255 referências. Após a leitura dos títulos e resumos, e considerados os nove artigos obtidos nas listas de referências, 88 textos foram conduzidos para leitura integral. Com 39 exclusões (não utilizaram modelos de regressão: n = 18; adoção do termo comportamento sedentário enquanto ausência da atividade física: n = 17; estudos que não verificaram o comportamento sedentário: n = 2; faixa etária fora dos três aos 19 anos: n = 1; pesquisa não conduzida no País: n = 1), 49 artigos foram considerados para elaboração da síntese descritiva (Figura).

**Figura:** Fluxograma da revisão sistemática.

Ao todo, 38 amostras distintas foram verificadas nos 49 artigos que compuseram a síntese, visto que distintos artigos avaliaram uma mesma amostra (o estado de Santa Catarina^63^ ^,^ ^64^, e as cidades: João Pessoa^61^ ^,^ ^62^, Maringá^13^ ^-^ ^16^, Pelotas^20^ ^-^ ^22^ ^,^ ^24^, Pernambuco^68^ ^,^ ^69^, Presidente Prudente^29^ ^-^ ^31^). No total, as amostras variaram entre 276^12^ e 109.104^56^ participantes, com maior percentual de meninas em 23 delas (60,5%). Apesar da distinção entre o número de artigos incluídos na síntese e o número total de amostras encontradas, as demais análises desta revisão foram fundamentadas no conjunto dos 49 artigos incluídos, respeitando seu caráter descritivo.

Ainda na Tabela 1, por localização geográfica, viu-se a predominância dos artigos produzidos na região Sul do País (n = 20; 40,8%), seguidos pelos artigos desenvolvidos nas regiões Sudeste e Nordeste, com 11 cada (22,4% para cada região). Nesse conjunto, destacam-se duas publicações de representatividade nacional, baseadas nos dados da PeNSE, que envolve adolescentes de todas as capitais do País e do Distrito Federal^7^ ^,^ ^56^. Apenas oito artigos tiveram prazo final de coleta dos dados anterior ao ano de 2005 (16,3%), e o comportamento sedentário foi a variável de desfecho em 13 artigos (26,5%).

**Tabela 1:** Características descritivas dos estudos incluídos.

Referência	Local (ano da coleta)	Amostra	Idade	%F	Var
Alves et al.^2^ (2012)	Salvador, BA (2006)	803	10-14	50,6	E
Balaban et al.^4^ (2010)	Juazeiro do Norte, Recife, PE e São Paulo, SP (2003-2004)	366	2-6	51,9	E
Beck et al.^5^ (2014)	Três de Maio, RS (2006)	660	14-19	52,0	E
Camelo et al.^7^ (2012)	Todas as capitais do País (2009)	59.809	9º ano EF	52,7	D
Campagnolo et al.^8^ (2008)	São Leopoldo, RS (2002, 2003)	810	10-19	59,4	E
Costa et al.^11^ (2011)	Florianópolis, SC (2002)	2.195	7-10	48,8	E
Crispim et al.^12^ (2013)	Goiânia, GO (2011-2012)	276	2-4	47,5	E
de Moraes et al.^13^ (2009)	Maringá, PR (2007)^a^	991	14-18	54,5	E
de Moraes et al.^14^ (2012)	Maringá, PR (2007)^a^	991	14-18	54,5	E
de Moraes; Falcão^15^ (2013)	Maringá, PR (2007)^a^	991	14-18	54,5	E
de Moraes et al.^16^ (2013)	Maringá, PR (2007)^a^	991	14-18	54,5	E
de Vitta et al.^17^ (2011)	Bauru, SP (2007)	1.236	11-14	51,8	E
de Vitta et al.^18^ (2014)	Bauru, SP (2009)	524	5ª-8ª série EF	46,9	E
Dias et al.^19^ (2014)	Cuiabá, MT (2009-2011)	1.716	10-17	49,3	D
Dumith et al.^20^ (2010)	Pelotas, RS (2004-2005)^b^	4.431	11	nd	D
Dumith et al.^21^ (2012)	Pelotas, RS (2008)^b^	4.118	15	49,9	D
Dumith et al.^22^ (2012)	Pelotas, RS (2008)^b^	4.120	15	49,9	E
Duncan et al.^23^ (2011)	Região oeste do Estado de São Paulo (nd)	3.397	7-18	53,0	E
Duquia et al.^24^ (2008)	Pelotas, RS (2004, 2005)^b^	4.452	11	50,8	E
Dutra et al.^25^ (2006)	Pelotas, RS (2003)	810	10-19	49,7	E
Enes; Slater^26^ (2013)	Piracicaba, SP (2004-2005)	431	10-13	56,0	E
Faria et al.^27^ (2014)	Viçosa, MG (nd)	800	10-19	51,2	E
Farias Jr. et al.^28^ (2012)	João Pessoa, PB (2009)	2.859	14-19	57,8	E
Fernandes et al.^29^ (2008)	Presidente Prudente, SP (2007)^c^	1.752	11-17	53,7	E
Fernandes et al.^30^ (2011)	Presidente Prudente, SP (2007)^c^	1.630	11-17	54,0	D
Fernandes et al.^31^ (2011)	Presidente Prudente, SP (2007)^c^	1.779	11-17	nd	E
Guimarães et al.^36^ (2013)	Curitiba, PR (2012)	572	12-17	57,0	E
Hackenhaar et al.^37^ (2013)	Cuiabá, MT (2009-2011)	1.716	10-17	50,7	E
Lippo et al.^41^ (2010)	Recife, PE (nd)	597	15-19	49,4	E
Melo et al.^45^ (2011)	Estado de Pernambuco (2010)	4.207	14-19	59,8	D
Oliveira et al.^49^ (2010)	São Luís, MA (2005)	592	9-16	50,5	D
Petribú et al.^52^ (2011)	Caruaru, PE (2007)	600	15-20	62,5	E
Rech et al.^54^ (2010)	Morro Reuter e Dois Irmãos, RS (2005)	1.442	7-12	50,0	E
Rech et al.^55^ (2013)	Caxias do Sul, RS (2011)	1.230	11-14	49,3	E
Rezende et al.^56^ (2014)	Todas as capitais do País (2012)	109.104	9º ano EF	nd	E
Ribeiro et al.^57^ (2003)	São Paulo, SP (2000)	446	7-10	52,0	E
Santos et al.^58^ (2013)	Uberaba, MG (2012)	649	9-12	52,1	D
Silva et al.^59^ (2011)	Florianópolis, SC (2007)	818	14-18	61,8	E
Silva Jr. et al.^60^ (2012)	Rio Branco, AC (2009)	741	14-18	54,1	E
Silva et al.^61^ (2007)	João Pessoa, PB (2005)^d^	1.570	7-12	48,5	D
Silva et al.^62^ (2007)	João Pessoa, PB (2005)^d^	1.570	7-12	48,5	E
Silva et al.^63^ (2008)	Estado de Santa Catarina (2001)^e^	5.028	15-19	59,4	E
Silva et al.^64^ (2009)	Estado de Santa Catarina (2001-2002)^e^	5.028	15-19	59,4	D
Silva et al.^65^ (2014)	Estado de Santa Catarina (2011)	6.529	15-19	57,8	D
Smith-Menezes et al.^66^ (2012)	Aracaju, SE (2007)	758	18	0	E
Suñé et al.^67^ (2006)	Capão da Canoa, RS (2004)	719	11-13	nd	E
Tassitano et al.^68^ (2009)	Estado de Pernambuco (2006)^f^	4.210	14-19	59,8	E
Tenório et al.^69^ (2010)	Estado de Pernambuco (2006)^f^	4.210	15-19	59,8	D
Vasconcellos et al.^71^ (2013)	Niterói, RJ (2010)	328	10-18	67,1	E

%F: percentual do sexo feminino na amostra; Var: variável (como o comportamento sedentário foi analisado); Var-D: variável de desfecho; Var-E: variável de exposição; EF: ensino fundamental; nd: não divulgado

^a-f^ Publicações que se utilizaram de amostras similares.

A síntese desta revisão incluiu 43 estudos transversais, três casos-controles^4^ ^,^ ^41^ ^,^ ^57^ e três dados da coorte de Pelotas^21^ ^,^ ^22^ ^,^ ^26^ (Tabela 2). Sobre a medida do comportamento sedentário, prevaleceram as observações das exposições ao tempo de tela (n = 27; 55,1%) e ao tempo de televisão (n = 16; 32,6%). Quanto aos pontos de corte utilizados para caracterizar o tempo excessivo em comportamentos sedentários, o mais frequentemente utilizado nos artigos foi o de pelo menos duas horas diárias, adotado em 16 publicações (30,6%), considerando o tempo de: tela (n = 10); televisão (n = 4), computador (n = 1); ou tela e sentado (n = 1). Mesmo que todos os artigos incluídos tenham utilizado questionários para avaliação do comportamento sedentário, observou-se grande variação nos instrumentos utilizados. Em 17 artigos (34,7%), os autores não relataram se o instrumento utilizado passou por validação prévia ou, nos casos de instrumentos estrangeiros, se houve validação para uso em populações brasileiras.

**Tabela 2:** Características metodológicas dos estudos incluídos.

Referência	Tipo	Seleção	Pontos de corte	Instrumento de avaliação	Modelo de regressão/Medida de efeito
Alves et al.^2^ (2012)	TR	ale	Tela > 3,3h/sem	QDE^a,b^	Poisson/RP
Balaban et al.^4^ (2010)	CC	conv	TV > 5h/d	QDE^p,nv^	Logística/OR
Beck et al.^5^ (2014)	TR	ale	Tela h/sem	QDE^nv^	Linear/Coef.
Camelo et al.^7^ (2012)	TR	ale	TV > 2h/d	PeNSE 2008	Logística/OR
Campagnolo et al.^8^ (2008)	TR	ale	TV > 2h/d	7-Day Recall^nv^	Poisson/RP
Costa et al.^11^ (2011)	TR	ale	Tela > 2h/d	QDE	Poisson/RP
Crispim et al.^12^ (2013)	TR	ale	TV > 2h/d	QDE^p,nv^	Poisson/RP
de Moraes et al.^13^ (2009)	TR	ale	Tela > 4h/d	IPAQ curto	Poisson/RP
de Moraes et al.^14^ (2012)	TR	ale	Tela h/d	QDE	Linear/Coef.
de Moraes; Falcão^15^ (2013)	TR	ale	Tela > 4h/d	QDE	Poisson/RP
de Moraes et al.^16^ (2013)	TR	ale	Tela > 2h/d	QDE	Linear/Coef
de Vitta et al.^17^ (2011)	TR	ale	Tela > 2h/d	QDE^c,nv^	Logística/OR
de Vitta et al.^18^ (2014)	TR	ale	Tela h/d	QDE^c,nv^	Logística/OR
Dias et al.^19^ (2014)	TR	ale	Tela > 4h/d	QDE^nv^	Logística/OR
Dumith et al.^20^ (2010)	TR-CO	nasc 1993	Tela > 2h/d	QDE	Poisson/RP
Dumith et al.^21^ (2012)	CO	nasc 1993	Tela Freq	QDE	Linear/Coef.
Dumith et al.^22^ (2012)	CO	nasc 1993	Tela > 2h/d	QDE	Poisson/RR
Duncan et al.^23^ (2011)	TR	ale	PC h/d	QDE	Logística/OR
Duquia et al.^24^ (2008)	TR-CO	nasc 1993	Tela > 4h/d	QDE	Poisson/RP
Dutra et al.^25^ (2006)	TR	ale	TV > 4h/d	QDE	Poisson/RP
Enes; Slater^26^ (2013)	CO	ale	Tela h/d	Questionário de Berkey^d^	Linear/Coef.
Faria et al.^27^ (2014)	TR	conv	Sentado min/d	IPAQ curto	Logística/OR
Farias Jr. et al.^28^ (2012)	TR	ale	Tela > 2h/d	QDE^nv^	Poisson/RP
Fernandes et al.^29^ (2008)	TR	ale	TV Freq	Questionário de Baecke	Poisson/RP
Fernandes et al.^30^ (2011)	TR	ale	TV Freq	Questionário de Baecke	Logística/OR
Fernandes et al.^31^ (2011)	TR	ale	TV Freq	Questionário de Baecke	Poisson/RP
Guimarães et al.^36^ (2013)	TR	conv	Total	Questionário QASA	Logística/OR
Hackenhaar et al.^37^ (2013)	TR	conv	Tela > 4h/d	Questionário COMPAC	Poisson/RP
Lippo et al.^41^ (2010)	CC	conv	Tela > 1h/d	QDE^nv^	Logística/OR
Melo et al.^45^ (2011)	TR	ale	TV > 3h/d	Questionário GSHS-OMS	Logística/OR
Oliveira et al.^49^ (2010)	TR	ale	Tela > 2h/d	Inquérito AF Recordatório 24h^e^	Linear/Coef.
Petribú et al.^52^ (2011)	TR	ale	TV > 3h/d	Questionário COMPAC	Logística/RP
Rech et al.^54^ (2010)	TR	conv	TV > 3h/d	QDE	Logística/RP
Rech et al.^55^ (2013)	TR	ale	Tela > 3h/d	QDE^nv^	Logística/RP
Rezende et al.^56^ (2014)	TR	ale	Tela + Sentado 2h/d	PeNSE 2012	Poisson/RP
Ribeiro et al.^57^ (2003)	CC	ale	TV > 4h/d	QDE^p^	Logística/OR
Santos et al.^58^ (2013)	TR	ale	Tela Freq	Questionário Hábitos de Vida	Poisson/RP
Silva et al.^59^ (2011)	TR	ale	TV > 2h/d	QDE^nv^	Logística/OR
Silva Jr. et al.^60^ (2012)	TR	ale	PC > 2h/d	IPAQ curto	Poisson/RP
Silva et al.^61^ (2007)	TR	ale	TV Freq	QDE	Logística/OR
Silva et al.^62^ (2007)	TR	ale	Tela h/d	QDE	Poisson/RP
Silva et al.^63^ (2008)	TR	ale	Tela > 2h/d	Questionário COMPAC	Logística/OR
Silva et al.^64^ (2009)	TR	ale	Tela > 4h/d	Questionário COMPAC	Poisson/RP
Silva et al.^65^ (2014)	TR	ale	Tela > 2h/d	Questionário COMPAC	Poisson/RP
Smith-Menezes et al.^66^ (2012)	TR	ale	Tela > 2h/d	IPAQ curto	Poisson/RP
Suñé et al.^67^ (2006)	TR	ale	Total > 4h 30 min	QDE^nv^	Poisson/RP
Tassitano et al.^68^ (2009)	TR	ale	TV > 3h/d	Questionário GSHS-OMS	Logística/OR
Tenório et al.^69^ (2010)	TR	ale	TV > 3h/d	Questionário GSHS-OMS	Logística/OR
Vasconcellos et al.^71^ (2013)	TR	ale	Tela h/sem	Questionário de Pate et al.^f^	Logística/OR

TR: transversal; ale: seleção aleatória; Tela: compreensão das atividades de tela, como televisão, computador e videogame; h/sem: horas por semana; QDE: questionário desenvolvido para o estudo; RP: razão de prevalências; CC: caso-controle; conv: seleção por conveniência; TV: televisão; h/d: horas por dia; OR: odds ratio; Coef.: coeficiente; PeNSE: Pesquisa Nacional de Saúde do Escolar; PC: computador; RR: risco relativo; IPAQ: Questionário Internacional de Atividade Física; TR-CO: transversal aninhado a um estudo de coorte; nasc: nascimento; CO: coorte; Freq: frequência; min/d: minutos por dia; QASA: Adolescent Sedentary Activity Questionnaire; COMPAC: Comportamento do Adolescente Catarinense; AF: atividade física; GSHS-OMS: *Global School-Based Student Health Survey* – Organização Mundial da Saúde (GSHS-OMS).

^a^ Adaptado do *Global School-Based Student Health Survey* – Organização Mundial da Saúde (GSHS-OMS).

^b^ Adaptado do *Pro Children Cross-sectional survey* (CSS).

^c^ Baseado no estudo de Harreby et al., “Risk factors for low back pain in a cohort of 1389 Danish school children: an epidemiologic study (*Eur Spine J* 1999;8:444)”.

^d^ Baseado no estudo de Berey et al., “Activity, Dietary Intake, and Weight Changes in a Longitudinal Study of Preadolescent and Adolescent Boys and Girls” (*Pediatrics* 2000,105:e56).

^e^ Baseado no *Self Administred Physical Activity Checklist*.

^f^ Traduzido e validado por Barros; Nahas, 2003 “Medidas da atividade física: teoria e prática em diversos grupos populacionais”.

^nv^ O artigo não comunica validação prévia do instrumento (ou não comunica validação da ferramenta internacional em populações brasileiras).

^p^ Abordagem proximal.

Os artigos analisados apresentaram análises entre o comportamento sedentário e 31 variáveis distintas (Tabela 3). A maior parte dessas variáveis foram classificadas como comportamentais (n = 12), dando-se também destaque ao elevado número de variáveis pertencentes aos domínios demográficos ou socioeconômicos (n = 7) e agravos à saúde (n = 6).

**Tabela 3:** Síntese das relações entre as variáveis e elevados volumes de comportamento sedentário em crianças e adolescentes brasileiros.

Domínios e variáveis	Associação como fator de risco	Associação como fator de proteção	Sem associação
Socioeconômico			
Condição socioeconômica elevada	Tempo de tela:^19,20^; PC+VG:^65^	Tempo de TV:^65 (F)^	Tempo de tela:^49,58,64,66^; TV:^31^
Cor da pele	–	–	Tempo de tela:^20^; TV:^69^
Idade	Tempo de tela:^19 (associação com aumento da idade)^	Tempo de TV:^65,69 (associação com faixa etária de 17 a 19 anos)^; Tempo de PC+VG:^65 (associação com faixa etária de 17 a 19 anos)^	Tempo de tela:^49,58,64^
Morar com os pais	–	–	Tempo de TV:^69^
Maior escolaridade materna	–	–	Tempo de TV:^69^
Escolaridade	–	–	Tempo de tela:^66^
Sexo	–	Tempo de TV:^69 (F)^	Tempo de tela:^20,58^; Tempo de TV:^69 (SEM)^
Ambiental			
Gostar do bairro em que mora	–	–	Tempo de tela:^20^
Local de atividades^a^	–	–	Tempo de tela:^20^
Local de residência	Tempo de PC+VG:^65 (associação com residir em área urbano)^	Tempo de tela:^19 (associação com residir no interior), 65 (associação com residir em área rural)^; Tempo de TV:^65 (F, associação com residir em área urbana), 69 (associação com residir em área rural)^	Tempo de tela:^64^; Tempo de TV:^69^
Comportamental			
Percepção de bem-estar	Tempo de tela:^20 (adolescentes que relataram maiores níveis de tempo de tela não se classificaram no nível mais alto de felicidade)^	–	–
Tempo de sono	–	–	Tempo de tela:^58^
Afiliação religiosa	Tempo de TV:^45 (elevados níveis de tempo de TV foram observados em pessoas de religião católica, FDS)^	–	Tempo de TV:^45 (não foi encontrada associação entre tipo de religião e elevados níveis de TV, SEM)^
Prática religiosa	–	Tempo de TV:^45 (a prática religiosa se mostrou como fator protetor a níveis elevados de tempo de TV)^	–
Prática esportiva^b^	–	–	Tempo de TV:^29 (prática de esportes)^; Tempo de tela:^58 (frequentar escola de esportes)^
Menor nível de atividade física	Tempo de tela + Tempo total sentado:^56 (AFL)^; Tempo de tela:^19 (300 minutos por semana), 21 (AFL), 22 (AFL, F), 63 (300 minutos por semana, M)^; Tempo de TV:^41 (IPAQ)^	Tempo de TV:^11 (proteção em crianças com níveis inferiores a 2h/dia de TV pelo escore DAFA), 30 (proteção em meninas com níveis inferiores a 2h/dia de TV no FDS)^	Tempo de tela:^13,20,22,28,58,63 (F)^; Tempo de TV:^2^; Tempo de PC:^41^
Experimentação de bebidas alcoólicas	Tempo de tela:^19^	–	–
Estado civil	–	–	Tempo de tela:^66^
Deslocamento para escola	–	–	Tempo de tela:^61^
Consumo de frutas e verduras	–	–	Tempo de tela:^64^; Tempo de TV:^30^
*Bullying* (ser vítima e agressor)	Tempo de tela:^55^	–	–
Alimentos e bebidas calóricas	Tempo de tela:^14 (F consumo de proteínas)^; Tempo de TV:^7,30^	–	Tempo de tela:^14 (F consumo de alimentos calóricos)^
Ocupacional			
Dispensa total ou pouca participação nas aulas de EF	–	–	Tempo de tela + Tempo total sentado:^56^; Tempo de TV:^69^
Trabalho	–	–	Tempo de tela:^64,66^
Turno de aula	–	–	Tempo de tela:^64^
Agravos à saúde			
Pressão Arterial Sistêmica	–	–	Tempo de tela:^16, 21,58^; Tempo de TV:^12^
Colesterol total	Tempo de tela:^36^	–	Tempo total:^36^; Tempo de tela:^5^
Dor musculoesquelética (pescoço/ombros)	Tempo de TV:^18^; Tempo de PC:^18 (M)^	–	Tempo de PC:^18 (F)^
Dor lombar	Tempo de tela:^17^	–	–
Níveis elevados de peso	Tempo total:^36,67,71^; Tempo de tela:^19 (na adolescência), 20,21,37^; Tempo de TV:^8,25,52,54,57,62 (M)^; Tempo de PC:^23,60^	–	Tempo de tela:^15,19 (infância),24,26,36,58,63^; Tempo de TV:^4,31,59,61 (F),68^
Resistência à insulina	Tempo total sentado:^27^	–	–

PC: computador; VG: videogame; TV: televisão; F: sexo feminino; SEM: dias da semana; AFL: atividade física no tempo de lazer; M: sexo masculino; IPAQ: classificação de atividades físicas moderadas e vigorosas de acordo com o Questionário Internacional de Atividade Física (IPAQ); escore DAFA: classificação de ser mais ativo (atividades físicas leves, moderadas e vigorosas) segundo o escore DAFA; FDS: dias do final de semana; Total: tempo total em comportamentos sedentários; EF: educação física

^a^ Relações entre o comportamento sedentário e a realização das atividades diárias em locais fechados (e.g., casa) ou abertos (e.g., parque).

^b^ Relações entre níveis de comportamento sedentário e a prática esportiva, assim como a participação em escola de esportes.

Sobre o número de artigos e a frequência das associações entre a medida de comportamento sedentário e as variáveis de um ou mais domínios, o principal resultado encontrado se deu nas associações positivas entre volumes elevados de comportamento sedentário e “níveis elevados de peso corporal”, observadas em 15 de 27 artigos que avaliaram esta relação (55,5%). A maior parte dos resultados que identificaram associações estatisticamente positivas entre o comportamento sedentário e a variável em questão foi verificada em estudos que avaliaram os comportamentos de tela, seja por tipos mais específicos, como televisão e computador, ou pelo tempo total de tela. Sobre a medida de efeito desses estudos, oito associações estatisticamente positivas foram comunicadas por meio de razões de prevalências, seis por *odds ratios* e uma por coeficiente (β). Ressalta-se que duas dessas associações positivas entre volumes mais elevados de comportamento sedentário e “níveis elevados de peso corporal” são provenientes de estudos longitudinais (um caso-controle e uma coorte) (Tabela 3).

Como resultado secundário, 50,0% dos artigos mostraram associações estatisticamente positivas entre volumes elevados de comportamento sedentário e “baixos níveis de atividade física”. Assim como no resultado anterior, a maior parte desses trabalhos avaliou o comportamento sedentário pelos comportamentos de tela (total, n = 4 e televisão n = 3). Por tipo de estudo, duas dessas associações positivas foram observadas nos estudos da coorte de nascimento de Pelotas de 1993^21^ ^,^ ^2^ ^2^ (Tabela 3).

Mesmo que presentes em apenas três artigos cada, outras duas observações devem ser consideradas: associações positivas entre o maior tempo de tela e o consumo de alimentos ricos em calorias e gorduras (como lanches, biscoitos doces e embutidos e bebidas calóricas)^7^ ^,^ ^14^ ^,^ ^30^ e as associações de proteção contra o comportamento sedentário por morar em cidades do interior ou em zonas rurais^19^ ^,^ ^64^ ^,^ ^69^. Por outro lado, devido ao número reduzido de artigos e à distribuição similar entre a presença ou ausência de associações estatisticamente positivas, mantém-se a incerteza quanto às possíveis associações entre elevados níveis de comportamento sedentário e as variáveis “condição socioeconômica elevada” (n = 9 artigos), “idade” (n = 7 artigos) e “sexo” (n = 3 artigos) (Tabela 3).

## DISCUSSÃO

Os principais resultados desta revisão sistemática fundamentaram-se nos dados de 49 artigos que envolveram amostras de populações de crianças e adolescentes brasileiros. Nesse conjunto, destacou-se o grande número de pesquisas transversais, assim como a grande discrepância metodológica entre os artigos quanto aos tipos de comportamentos sedentários avaliados e pontos de corte utilizados para caracterizar o tempo excessivo em comportamentos sedentários. Os artigos analisados apresentaram maior consistência nas associações estatisticamente positivas entre tempo elevado de exposição ao comportamento sedentário e duas variáveis: (i) níveis elevados de peso corporal e (ii) baixos níveis de atividade física.

Sobre os aspectos metodológicos, o presente estudo identificou a inexistência de padrões na avaliação do comportamento sedentário em crianças e adolescentes, seja por diferentes formas de operacionalizar esses comportamentos (e.g., tempo de tela, tempo sentado, tempo de televisão) ou pelos métodos e ferramentas que são utilizados na sua avaliação (questionários internacionais, questionários nacionais, ou mesmo questionários desenvolvidos para os próprios fins)^9^ ^,^ ^51^. Uma vez que todos os estudos utilizaram questionários para a obtenção das medidas de comportamento sedentário, um dos principais desafios às futuras pesquisas é a incorporação de medidas objetivas em seus métodos (e.g., por meio dos sensores de movimento, como o ActiPal e acelerômetros), de modo que esta estratégia venha a complementar os dados do autorrelato, controlando o viés de memória, tal como observado em estudo com adultos^39^.

Doze publicações (24,5%) não reportaram a evidência de validade do instrumento utilizado. Esses achados reforçam a observação de Atkin et al.^3^, que destacam a carência de bons instrumentos para avaliar o comportamento sedentário em pesquisas epidemiológicas. É importante que futuras pesquisas, além de relatarem o processo de validação de seus questionários ou apresentarem as referências que embasam tal procedimento, façam uso de questionários validados.

Corroborando os achados de uma revisão prévia^70^, a maioria dos estudos encontrados caracteriza o comportamento sedentário como medida do tempo de televisão ou do tempo de tela, que combina os indicadores tempo de televisão, computador e videogame. Contudo, esses comportamentos representam apenas parte do tempo total que crianças e adolescentes ficam sentados ao longo do dia enquanto estão acordados. Complementarmente, os estudos incluídos na presente revisão apresentaram alta heterogeneidade entre os pontos de cortes adotados para definir o tempo excessivo de exposição ao comportamento sedentário. Observou-se que o ponto de corte de duas horas diárias foi o mais frequente entre os estudos incluídos, utilizado em 16 artigos. Ainda que as pesquisas brasileiras não tenham esclarecido os riscos à saúde do volume de tempo permanecido em comportamentos sedentários, revisão sistemática de estudos internacionais identificou associações estatisticamente significantes entre exposições de pelo menos duas horas e excesso de peso, baixa aptidão física, baixa autoestima e sociabilização, e baixo desempenho acadêmico^70^.

Sobre os modelos de regressão adotados, em 22 artigos (21 transversais e uma coorte) se utilizou a regressão de Poisson e em 21 artigos (19 transversais e dois casos-controles) se utilizou a regressão logística. Em seis artigos (quatro transversais e duas coortes) as análises foram realizadas por meio do modelo de regressão linear.

As associações positivas verificadas entre maior tempo de comportamento sedentário e níveis elevados de peso corporal corroboraram as sínteses de revisões que envolveram crianças e adolescentes de distintos países, independentemente de sua condição socioeconômica^43^ ^,^ ^47^, e uma síntese de estudos prospectivos conduzidos em países de renda alta, como Austrália, Canadá e Estados Unidos^48^. Tal como se verificou no presente artigo, é importante acenar que estas evidências estão, em grande parte, fundamentadas nos resultados de artigos que avaliaram comportamentos de tela, com enfoque particular no tempo de televisão.

Já as associações positivas verificadas entre maior tempo de comportamento sedentário e baixos níveis de atividade física concordam com estudo conduzido em países de renda alta que mostrou relações negativas entre elevado tempo de tela e prática de atividades físicas moderadas e vigorosas^44^. A principal recomendação de atividade física para ganhos em saúde preconiza a realização de atividades físicas com intensidades moderadas e vigorosas, desconsiderando as atividades físicas leves^a^. Correlações inversas mais fortes têm sido encontradas entre comportamento sedentário e atividades físicas leves^39^. Para evitar longos períodos de tempo ininterruptos em comportamentos sedentários, propõe-se interrupções (*“breaks”*) constantes nas atividades caracterizadas como sedentárias (< 1,5 MET), por inserção de atividades com gasto energético mais elevado^38^. Apesar de meta-análise recente ressaltar o potencial do ambiente escolar para a realização dessas estratégias em crianças e adolescentes^33^, nenhuma das intervenções incluídas foi conduzida no Brasil, o que traz à tona a necessidade desses estudos na agenda nacional de pesquisa.

Apesar de observadas em menor número de publicações, foram encontradas associações positivas entre maior tempo em comportamento sedentário e consumo de alimentos e bebidas calóricas e as associações positivas entre menor tempo em comportamento sedentário e residência em cidades do interior ou em zonas rurais. Em relação à primeira, os dados da presente síntese corroboraram os achados de recentes estudos internacionais que mostraram associações entre volumes prolongados de tempo de tela (≥ 20 horas semanais) e alto consumo de alimentos calóricos (lanches, doces e biscoitos) e de refrigerantes^6^ ^,^ ^34^ ^,^ ^42^. Quanto ao efeito protetor de residir no interior ou em zonas rurais, acredita-se que crianças e adolescentes que vivem nesses locais possam dispor de maiores oportunidades para a prática de atividade física. As condições mais favoráveis de segurança e tráfego possibilitariam a prática de brincadeiras de forma mais livre nas comunidades e também o uso mais ampliado da bicicleta^19^ ^,^ ^64^. Também diminuiria comportamentos sedentários como atividades de tela no tempo de lazer e assistir televisão por períodos de tempo prolongados. Entretanto, como ainda é pequeno o número de estudos nacionais disponíveis que averiguaram a ocorrência dessas associações, mais pesquisas são necessárias.

Esta revisão sistemática mostrou algumas lacunas entre as pesquisas brasileiras sobre o comportamento sedentário, como o alto número de estudos transversais incluídos, em comparação ao número reduzido de pesquisas longitudinais^53^. Uma vez que o delineamento de estudo transversal não estabelece precedência entre as variáveis de exposição e de desfecho, problemas relacionados à causalidade reversa podem ocorrer, como no caso do principal achado da pesquisa, em que não é possível verificar a sequência cronológica entre os comportamentos sedentários e os níveis de peso corporal e de prática de atividade física das crianças e adolescentes. Além disso, a ausência de estudos longitudinais limita inferências sobre as relações dose-resposta entre o comportamento sedentário e as variáveis encontradas. Outra limitação foi a alta heterogeneidade dos artigos incluídos quanto às formas utilizadas para avaliar o comportamento sedentário (tempos de tela, de televisão, de computador, sentado e total), aos pontos de corte adotados e aos instrumentos utilizados para sua avaliação.

A inclusão de todos os estudos com repetidas amostras pode ser considerada uma limitação potencial desta revisão sistemática. Tal opção pode ter superestimado os resultados de comparação das relações do comportamento sedentário com outros desfechos ou exposições, como os níveis elevados de peso corporal ou baixos níveis de atividade física. No entanto, duas opções metodológicas conduziram à inclusão desses artigos: o caráter descritivo da presente revisão, que buscou oferecer um panorama geral das variáveis associadas ao comportamento sedentário em crianças e adolescentes brasileiros e o não planejamento da realização de meta-análise.

Com base nos dados identificados na presente revisão, algumas conclusões podem ser tomadas. Quanto aos aspectos metodológicos, ainda há poucos estudos que mensuram o comportamento sedentário para além da medida do tempo de tela (televisão, computador, videogame). Novos estudos podem envolver medidas de tempo nos aparelhos celulares e *tablets*, assim como medidas em outros domínios, como tempo sentado no deslocamento ou no ambiente escolar. Outro ponto é a necessidade do uso de questionários validados, como recurso paralelo às mensurações objetivas, de modo que tal procedimento fortaleça as informações sobre a associação entre tempo despendido em comportamentos sedentários e diferentes desfechos. Em vista da predominância dos estudos transversais, são necessárias pesquisas com abordagens longitudinais, sobretudo voltados a estabelecer a sequência cronológica dos eventos e a relação dose-reposta. Sobre os resultados disponíveis, os estudos realizados com amostras de crianças e adolescentes brasileiros mostraram principalmente associações entre elevado volume de comportamento sedentário e “níveis elevados de peso corporal” e “baixos níveis de atividade física” em distintas regiões do território brasileiro.

## Footnotes

a World Health Organization. *Global recommendations on physical activity for health*. Geneva: World Health Organization; 2010.
